# The potential role of CT enterography and gastrointestinal ultrasound in the evaluation of anti-tubercular therapy response of intestinal tuberculosis: a retrospective study

**DOI:** 10.1186/s12876-019-1030-0

**Published:** 2019-06-26

**Authors:** Li Ma, Qingli Zhu, Yue Li, Wenbo Li, Xuan Wang, Wei Liu, Jianchu Li, Yuxin Jiang

**Affiliations:** 1 Department of Ultrasound, Peking Union Medical College Hospital, Chinese Academy of Medical Sciences, Beijing, China; 2 Department of Gastroenterology, Peking Union Medical College Hospital, Chinese Academy of Medical Sciences, Beijing, China; 3 Department of Radiology, Peking Union Medical College Hospital, Chinese Academy of Medical Sciences, Beijing, China

**Keywords:** Intestinal tuberculosis, CT enterography, Gastrointestinal ultrasound, Anti-tubercular therapy, Treatment response evaluation

## Abstract

**Background:**

Accurate evaluation of anti-tubercular therapy (ATT) responses is crucial for both diagnosis and treatment of intestinal tuberculosis (ITB). Little is known about the role of cross-sectional imaging techniques in ITB follow-up assessment. We aimed to investigate the accuracy of cross-sectional imaging modalities, CT enterography (CTE) and gastrointestinal ultrasound (GIUS), in the evaluation of ATT responses in ITB patients.

**Methods:**

Patients diagnosed with ITB and followed up by CTE and/or GIUS were retrospectively searched in the databases. Clinical, imaging, laboratory and endoscopic data were collected at baseline and the first follow-up visit. Responses were graded as good, partial and no response based on protocols described in the literature and by our institution. CTE evaluation was based on changes in the lesion area, mural thickness, enhancement patterns and lymph nodes, while GIUS evaluation was based on changes in bowel wall morphology and the Limberg score. Clinical evaluation was used as the gold-standard evaluation method, which was determined by a comprehensive impression of endoscopic changes along with symptomatic improvement and laboratory tests, with imaging results masked.

**Results:**

Twenty patients with ITB were enrolled in our study. The first follow-up time was from 2 to 12 months (average 6 months). According to the gold standard evaluation, 11 patients were evaluated as having a good ATT response, while 9 had a partial response. A total of 18 patients were followed up by CTE, while 7 were followed up by GIUS, depending on medical and/or financial considerations. The accuracy of CTE and GIUS was 83% (15/18) and 85.7% (6/7), respectively. The sensitivity, specificity, PPV and NPV of CTE were 88.9, 77.8, 80 and 87.5%, respectively. Moreover, the sensitivity, specificity, PPV and NPV of GIUS were 100, 50, 83.3 and 100%, respectively. By combining the results of CTE and GIUS results, the overall accuracy was 90%, with sensitivity and specificity of 91.7 and 87.5%, respectively.

**Conclusion:**

To our knowledge, this is the first study exploring the accuracy of the cross-sectional imaging modalities CTE/GIUS in the evaluation of ATT responses. Our results indicated their promising application prospect in clinical practice as a non-invasive and cost-effective approach.

**Electronic supplementary material:**

The online version of this article (10.1186/s12876-019-1030-0) contains supplementary material, which is available to authorized users.

## Background

To date, tuberculosis (TB) remains a global infectious disease with high prevalence in developing countries and immunocompromised populations [1]. Approximately one-third of the worldwide population has been infected with *M. tuberculosis* [2], and nearly 10 million new TB cases are reported every year [3]. Intestinal tuberculosis (ITB) accounts for approximately 5% of all TB cases, mimicking a number of chronic gastrointestinal disorders, such as Crohn’s disease (CD), intestinal lymphoma and Behcet’s disease [4]. ITB can result in long-term gastrointestinal symptoms, and has been shown to greatly compromise quality of life [5].

Current diagnostic and therapeutic regimens of ITB greatly rely on the evaluation of the responses to anti-tubercular therapy (ATT). Several approaches for ITB diagnosis exist, including acid-fast bacilli, distinctive pathological epithelioid granuloma with caseous necrosis and isolation of *M.tuberculosis*, however, the sensitivity of these tests is low [6–9]. The diagnostic value of TB-PCR and T.spot. TB remains controversial. Under this condition, a more practical way to diagnose ITB is to evaluate the responses of diagnostic ATT in patients with suspected ITB symptoms and endoscopic manifestations, which has become a widely-used method for ITB diagnosis [10–12]. With regard to therapy, the standard course of ATT can be as long as 6 to 12 months. In addition, adverse drug reactions of ATT are common, and may therefore lower the compliance of patients. Therefore, in some cases, careful consideration of efficacy and adverse effects is required to make decisions regarding drug suspension or withdrawal. Thus, reliable evaluation of ATT responses is urgently required for both diagnosis and treatment.

Current widely-used method to evaluate the response to ATT by gastroenterologists is a comprehensive judgment, consisting of colonoscopy responses, clinical symptomatic recovery and changes in serological inflammatory markers (e.g. erythrocyte sedimentation rate (ESR) and hypersensitive C-reaction protein (hsCRP)), with colonoscopy changes as the most important consideration [13]. However, there are several limitations: except for terminal ileum, colonoscopy cannot reach most of the small intestine, which is also a common lesion site for ITB; the bowel preparation of the colonoscopy is intolerable for some patients with severe bowel lesions, and may worsen the disease and cause complications; in some cases with intestinal stenosis, endoscopic views can be largely restricted, and may therefore compromise the integrity of assessment; in some other patients with milder disease, the repeated painful experience may lower their follow-up compliance.

In recent years, cross-sectional imaging modalities such as CT enterography (CTE) and gastrointestinal ultrasound (GIUS) have been used in ITB for diagnosis and disease assessment [10, 14–16]. The role of CTE/GIUS in the evaluation of ATT responses of ITB is unknown. In this study, we evaluated the sensitivity and specificity of CTE and GIUS in the assessment of responses to ATT.

## Methods

### Patients and study design

This is a retrospective cohort study. The inpatient database from September 2015 to April 2019 of Peking Union Medical College Hospital, Beijing, China was reviewed. The inclusion criteria were: 1) Diagnosis of ITB, which must meet at least one of the following criteria: a. a characteristic epithelioid granuloma with caseous necrosis in biopsy samples of the intestine; b. positive results for *M.tuberculosis* culture or acid-fast bacilli (AFB) smear in intestinal tissues; c. typical clinical, imaging and endoscopic manifestations, plus active pulmonary TB or positive response to ATT for at least 2–8 weeks during follow-up. 2) Patients were started with ATT during hospitalization, then regularly followed up in our clinic after discharge, with full records of clinical, laboratory, imaging and endoscopic data before and after ATT. The exclusion criteria included: 1) Other accompanied chronic bowel diseases, such as malignant intestinal tumors, Crohn’s disease, Behcet’s disease. 2) The time interval between the colonoscopy and imaging examinations was longer than 2 months. 3) Discontinuance of ATT, or received surgery during follow-up period. Informed consent was obtained from all individual participants included in this study.

Baseline characteristics including demographic information, clinical course, past and family history, laboratory tests, imaging data, colonoscopy and pathological findings of the enrolled patients were collected. In general, patients were scheduled for the first follow-up visit within 6 months after therapy initiation, during which an overall re-evaluation of clinical symptoms, laboratory tests, CTE/GIUS and colonoscopy were performed. Another follow-up visit was usually paid when ATT ended. The evaluation results of the first follow-up visit were chosen for analysis in our study. Owing to patients’ personal reasons and disease conditions, the first follow-up time varied from 2 to 12 months (Additional file 1: Table S1).

### Anti-tubercular therapy

A standard combined chemotherapy composed of rifampicin (10 mg/kg), isoniazid (5 mg/kg), pyrazinamide (20–25 mg/kg) and ethambutol (15–20 mg/kg) for 2 months, followed by isoniazid and rifampicin, with/without adjuvant drugs were prescribed to ITB patients [17]. The total therapy duration is 6–12 months, depending on disease severity and ATT response of each patient (Additional file 1: Table S1).

### Clinical evaluation

Two gastroenterologists with more than ten years of experience independently reviewed colonoscopy images and reports, along with clinical and laboratory records. The evaluation was classified as good response, partial response and no response, according to the literature and protocol in our institution [18, 19]. The detailed criteria were shown in Table 1. If the two reviewers had different opinions, a third experienced gastroenterologist would review the data again and make a final judgment. The gastroenterologists were uninformed of the cross-sectional imaging information.

**Table 1:** Classification criteria of clinical, CTE and GIUS evaluations

	Good response	Partial response	No response
Clinical evaluation	Healing of all ulcers; or complete mucosal healing^a^ of over 50% lesion area compared to the baseline colonoscopy; or mucosal healing of less than 50% lesion area, but over 50% decrease of hsCRP levels, plus over 50% symptom improvement [19]	Improvements in some manifestations but cannot reach the “good response” criteria	No improvement in any manifestations
CTE evaluation	Lesion area decreased over 50% compared to previous CTE; or lesion area decreased less than 50%, plus a significant decrease in bowel wall thickness, less bowel wall enhancement and smaller lymph nodes	Improvements in some manifestations but cannot reach the “good response” criteria^b^	No improvement in any CTE manifestations
GIUS evaluation	Lesion area decreased over 50% compared to previous GIUS; or lesion area decreased less than 50%, plus Limberg score decreased at least two grades [20]	Improvements in some manifestations but cannot reach the “good response” criteria	No improvement in any US manifestations

^a^Complete mucosal healing is defined as completely normal appearance or residual fibrotic strictures of the mucosa

^b^Shrinkage of lymph nodes alone should not be considered as a sign of an effective therapeutic response

### Cross-sectional imaging examination and interpretation

Ultrasound was performed by an experienced radiologist using Philips iU22 (Philips,Bothell,WA,USA) with convex (C5–2) and linear (L9–3) transducers. Patients were fasted for at least 8 h before examination. A thorough scanning of the intestine was performed from duodenum to sigmoid. CTE was performed using a 16-slice MDCT scanner (GE LightSpeed Pro; GE Healthcare, Waukesha, WI). Bowel cleanse was performed before CTE exanimation. Patients ingested 1500 ml of mannitol followed by 500 ml water over 60 min prior to the CTE examination for bowel extension. Afterwards, patients were scanned during the enteric phase (peak small bowel enhancement) 50s after intravenous injection of iodinated contrast agent. Multi-planar images were reconstructed with high spatial resolution (slice thickness ≤ 3 mm).

For the cross-sectional imaging evaluation, two radiologists with more than ten years of experience individually reviewed the CTE and US images. If the two reviewers had different opinions, a third experienced radiologist would review the imaging data again and make a final judgment. The radiologists were unaware of the clinical, laboratory, endoscopic information, including the diagnosis, and other forms of imaging findings except for the reviewed ones.

For CTE, six manifestations, including the location of lesions, mural thickening, enlarged lymph nodes, bowel wall contrast-enhancement and complications (ascites, obstruction and perforation) were assessed [10]. The degree of the disease severity of the intestine was classified according to Kalra et al. [21] and Macari et al. [22]. Briefly, the bowel lesion area was graded as focal (≤5 cm), segmental (5-40 cm) or diffuse (>40 cm); the wall thickening was defined as normal (< 3 mm), mild (3–4 mm), moderate (5–9 mm), and marked (≥10 mm) [21, 22]. Lymphadenopathy was defined as a short axis over 10 mm in the upper abdomen, and over 15 mm in the pelvis [23]. Based on the protocol in our institution summarized in Table 1, CTE evaluation was classified as good response, partial response and no response.

For GIUS, bowel wall thickness, bowel wall morphology and bowel wall vascularity were examined [14]. Thickness of the bowel wall combined with vascularity were graded according to the Limberg score, a scoring system widely used for Crohn’s disease [20, 24]: grade 0, a bowel wall thickness of 3-4 mm without vascularization; grade 1, bowel wall thickening (> 4 mm) without vascularization; grade 2, bowel wall thickening with short stretches of vascularity; grade 3, bowel wall thickening with longer stretches of vascularity; grade 4, bowel wall thickening with vascularity into the mesentery [20]. The morphology of five layers of intestine was evaluated as “clear” and “vague”. Since ITB mostly affect the ileocecal area, the size and the depth of the ulcers in the ileocecal area were also carefully examined and taken as an independent manifestation. Other common manifestations of ITB, including intramural and extramural abscesses, fistula, mesenteric thickening, enlarged mesenteric lymph nodes were not shown in our study [14]. Based on the protocol in our institution summarized in Table 1, GIUS evaluation was classified as good response, partial response and no response.

The combined evaluation of CTE plus GIUS was determined by one radiologist, who comprehensively assessed CTE and GIUS images based on the criteria shown in Table 1.

### Statistics

Categorized data were assessed with chi-square test. A *P* value < 0.05 was considered significant. The specificity, sensitivity, positive predictive value, negative predictive value and accuracy rate were calculated. Statistical analysis was done using the SPSS Statistics software (v23).

## Results

### Study population

In this study, 28 patients who met the inclusion criteria were included in our database. Eight cases were excluded for the following reasons: 4 patients were suffering from gastrointestinal comorbidities, such as suspicious tumors or Behcet’s disease; 3 patients exhibited time intervals between clinical and cross-sectional imaging evaluations that were too long, and 1 patient underwent colectomy during the follow-up period. Therefore, 20 patients were enrolled in our study. A total of 3 patients were diagnosed pathologically, and 17 patients were diagnosed clinically after showing positive responses to ATT (good or partial response). Eighteen patients used CTE for follow-up, while 7 used GIUS for follow-up, therefore, 5 patients were assessed by both CTE and GIUS. The average follow-up time was 6.1 months (range: 2–12 months). The follow-up time and ATT duration are presented in detail in Additional file 1: Table S1.

### Clinical characteristics at baseline

Baseline characteristics of all enrolled patients are shown in Table 2. The study population included 14 male and 6 female patients; the average age was 43 years old (range: 16–64). The most frequent symptom was abdominal pain (85%), followed by weight loss (60%), diarrhea (50%), fever (50%) and night sweats (25%). Sites of intestinal involvement were 2.6 on average per patient. The distal ileum was the most commonly involved site (76%), followed by other small intestine (distal ileum excluded) (55%), ascending colon (50%), ileocecal area (35%), transverse colon (25%), and sigmoid colon (25%). Three patients had active pulmonary TB (15%), while 4 patients had a TB contact history (20%). The baseline clinical presentations in our enrolled patients were consistent with the earlier reports of ITB [16, 25, 26].

**Table 2:** Clinical and demographic characteristics of the enrolled patients

Characteristics^a^	Patients (*n* = 20)
Gender (male:female)	14:6
Median age, yr. (range)	43 (16–64)
Symptoms	
Abdominal pain	17 (85%)
Diarrhea	10 (50%)
Weight loss	12 (60%)
Night sweats	5 (25%)
Fever	10 (50%)
Sites of involvement per patient^a^	2.6 (1–5)
Locations of lesions	
Distal ileum	15 (75%)
Ileocecal area	7 (35%)
Other small intestine (distal ileum excluded)	11 (55%)
Ascending colon	10 (50%)
Transverse colon	5 (25%)
Descending colon	0
Sigmoid colon	5 (25%)
Rectum	0
Active pulmonary TB	3 (15%)
TB contact history	4 (20%)

^a^Sites of involvement were summarized from endoscopy and imaging results

### Serological hsCRP levels before and after ATT

The serological high-sensitivity C-reactive protein (hsCRP) levels before and after ATT are presented in Table 3. At baseline, 90% (18/20) patients showed elevated hsCRP levels. At follow-up visits, hsCRP levels were declined in 14 patients, remained at a plateau level in 3 patients, and were increased in 1 patient.

**Table 3:** Serological hsCRP levels before and after ATT

hsCRP levels	Patient No.
Elevated at baseline	18
Decreased after ATT	14
Plateaued after ATT	3
Increased after ATT	1
Normal at baseline	2

### Cross-sectional imaging manifestations

Cross-sectional imaging manifestations before and after ATT are summarized in Table 4. CTE findings included mural thickening, abnormal bowel wall enhancement, enlarged lymph nodes and fistulas. GIUS findings included bowel wall thickness, bowel wall morphology, vascularity, ileocecal ulcers, fistulas and strictures. After ATT, significant recovery patterns were observed. The bowel wall thickness detected by GIUS decreased by 60% before and after ATT and was statistically significant (*P* < 0.01).

**Table 4:** Cross-sectional imaging manifestations of ITB before and after ATT

Cross-sectional imaging modalities	Before ATT	After ATT	*P*
CTE			
Lesion Ranges	None: 2/18	None: 5/18	
	Focal: 6/18	Focal: 6/18	
	Segmental: 8/18	Segmental: 5/18	
	Diffuse^a^: 2/18	Diffuse: 2/18	
Thickened bowel wall	Normal: 0/18	Normal: 5/18	
	Mild: 2/18	Mild: 6/18	
	Moderate: 10/18	Moderate: 5/18	
	Marked: 6/18	Marked: 2/18	
Enlarged mesenteric lymph nodes	11/18	4/18	
Lymph nodes with necrosis or calcification	4/18	4/18	
Abnormal enhancement	14/18	4/18	
Fistulas	2/18	2/18	
GIUS			
Bowel wall thickness [cm; median (range)]	1.0 (0.5–1.6)	0.6 (0.4–1.1)	< 0.01
Bowel wall structure	Clear: 2/7	Clear: 5/7	
	Vague: 5/7	Vague: 2/7	
Vascularity	No vascularity: 1/7	No vascularity: 1/7	
	Short stretches: 1/7	Short stretches: 1/7	
	Long stretches: 2/7	Long stretches: 4/7	
	Into the mesentery: 3/7	Into the mesentery: 0/7	
Ileocecal ulcers	4/7	2/7	
Fistulas	2/7	0/7	
Strictures	2/7	2/7	
Enlarged mesenteric lymph nodes	0/7	0/7	

^a^Diffuse lesions only occurred in the small intestine

### Evaluation of ATT response

**Fig. 1:**
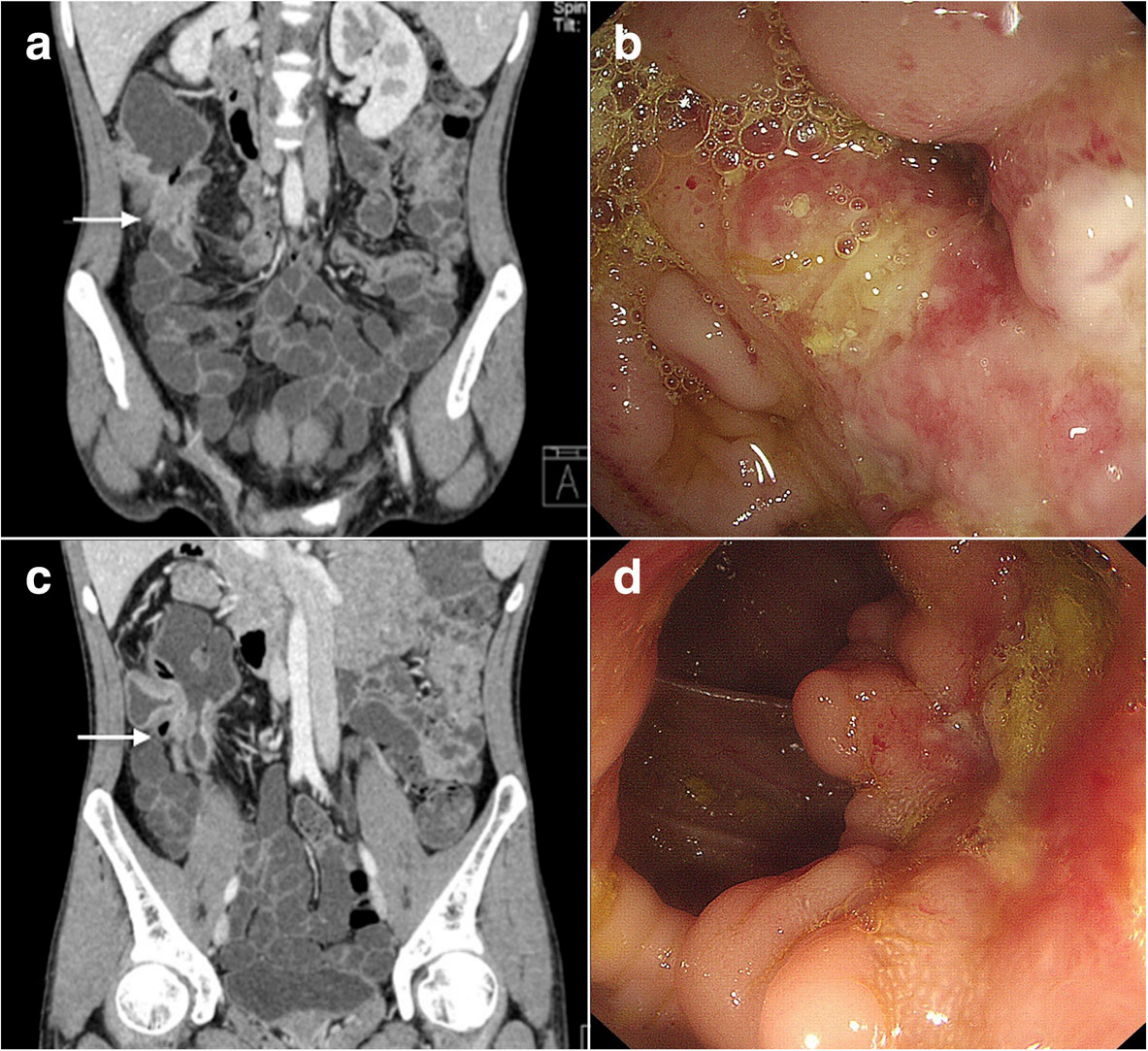
CTE and endoscopic manifestations before and after ATT. A 26-year-old male evaluated as having a “good response” by both CTE and clinical evaluation methods. **a** CTE image of the ileocecal area before ATT showing bowel wall thickening, stenosis and abnormal contrast-enhancement (arrow). **b** Colonoscopy image of the ileocecal area before ATT showing large ulcers and stenosis. **c** CTE image of the same area after 3 months of ATT, showing decreased lesion range, bowel wall thickness and stenosis. **d** Colonoscopy image after ATT, showing a significant shrinkage of ulcers and stenosis, corresponding to the CTE image

**Fig. 2:**
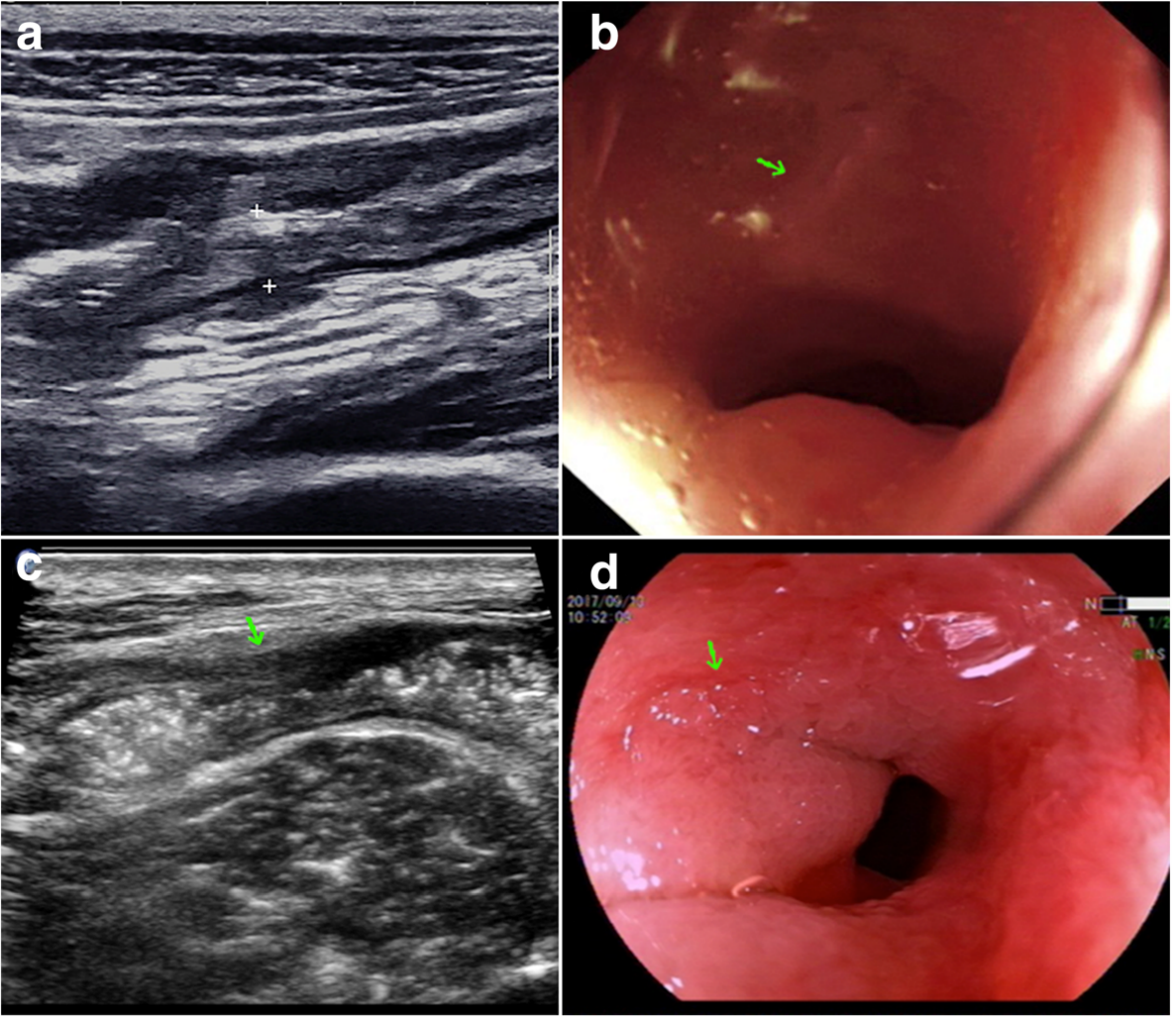
GIUS and endoscopic manifestations before and after ATT. A 62-year-old male evaluated as having a “good response” by both GIUS and clinical evaluation methods. **a** GIUS image of the terminal ileum before ATT showing stiff and thickened (10 mm) bowel walls (between crosses). **b** Colonoscopy image showing an annular ulcer in the terminal ileum before ATT (arrow). **c** GIUS image of the same area after 4 months of ATT showing normal echoic bowel wall (between crosses) and a localized stenosis (arrow). **d** Colonoscopy image after ATT showing mucosal healing and a stenotic scar (arrow)

Colonoscopy-based clinical evaluation was used as the gold-standard criteria. A total of 11 patients were graded as having a good response, and 9 patients had a partial response. No patients were non-responsive to ATT. Evaluation results of CTE and GIUS are listed in Table 5. CTE evaluated correctly in 15 of 18 (83%) patients. Comparable CTE and colonoscopy images before and after ATT of a representative patient are shown in Fig. 1. The patient was evaluated as having a “good response” by both CTE and GIUS. At baseline, CTE showed a thickened bowel wall, intestinal stenosis and hyperenhancement in the ileocecal area, while colonoscopy showed intestinal stenosis and ulcers in the corresponding area (Fig. 1a and b). After ATT, wall thickening and stenosis were notably recovered on CTE (Fig. 1c), while ulcers were smaller on colonoscopy (Fig. 1d). GIUS evaluated correctly in 6 of 7 (85.7%) patients. Comparable GIUS and colonoscopy images before and after ATT of a representative patient are shown in Fig. 2. The patient was evaluated as having a “good response” by both CTE and GIUS. At baseline, GIUS revealed the hypoechoic bowel wall with marked thickening (10 mm) at the terminal ileum (Fig. 2a). After 4 months of ATT, bowel wall thickness reduced to 5 mm with normal echogenicity (Fig. 2c) and vascularity (data not shown). Localized stenosis was present at the same site, which is a classical manifestation of healed ITB lesions. Correspondingly, an annular ulcer at the terminal ileum was shown on colonoscopy before ATT (Fig. 2b), which turned into scar stenosis after ATT (Fig. 2d). The sensitivity, specificity, positive and negative predictive values of CTE were 88.9, 77.8, 80 and 87.5% respectively. In addition, sensitivity, specificity, positive and negative predictive values of GIUS were 100, 50, 83.3 and 100%, respectively (Table 6). The overall accuracy rate of CTE + GIUS was 18/20 (90%), and sensitivity, specificity, positive, and negative predictive values were 91.7, 87.5, 91.7 and 83.3%, respectively, with a positive likelihood ratio of 7.3 (Table 6). The correlation of evaluation results between CTE/GIUS and colonoscopy is shown in Additional file 1: Table S2.

**Table 5:** CTE, GIUS and clinical evaluations of ATT response

Patient No.	Imaging Evaluation		Clinical Evaluation (Gold standard)
	CTE	GIUS	
1	Good Response	/	Good Response
2	Partial Response	/	Partial Response
3	Good Response	/	Good Response
4	Good Response	Good Response	Good Response
5	Good Response	/	Partial Response
6	Partial Response	/	Partial Response
7	Partial Response	/	Good Response
8	Partial Response	/	Partial Response
9	Partial Response	Partial Response	Partial Response
10	Good Response	Good Response	Good Response
11	No Response	Good Response	Partial Response
12	Good Response	/	Good Response
13	Good Response	/	Good Response
14	Partial Response	/	Partial Response
15	Good Response	Good Response	Good Response
16	/	Good Response	Good Response
17	/	Good Response	Good Response
18	Good Response	/	Good Response
19	Partial Response	/	Partial Response
20	Partial Response	/	Partial Response

**Table 6:** Sensitivity, specificity, positive predictive value, negative predictive value and accuracy of CTE and GIUS for evaluation the response to ATT in ITB

	Sensitivity	Specificity	Positive predictive value	Negative predictive value	Accuracy	LR+
CTE	88.9%	77.8%	80%	87.5%	80%	4
GIUS	100%	50%	83.3%	100%	85.7%	2
CTE + GIUS	91.7%	87.5%	91.7%	83.3%	90%	7.3

## Discussion

To the best of our knowledge, this is the first study in which the role of cross-sectional imaging techniques was explored in the evaluation of ATT responses in ITB patients. In our study, high sensitivity, specificity and accuracy were achieved for both CTE and GIUS, thereby suggesting that cross-sectional imaging modalities are a promising tool for the evaluation of treatment responses for clinical use.

Currently, the assessment of ATT responses in ITB patients includes symptomatic response assessment, serological markers and colonoscopy responses [27]. In general, symptomatic responses are subjective and non-standardized, and unparalleled to objective assessment (i.e. colonoscopy); therefore, they are unreliable indicators for response assessment [19]. Serum-CRP is a non-invasive method and declined CRP levels may reflect the effectiveness of ATT [27]. However, as a general indicator of inflammation, CRP lacks specificity to certain diseases in case of comorbidities. Moreover, as described by Sharma et al., several patients with abdominal tuberculosis had normal CRP levels before ATT treatment; therefore, the assessment could not be applied in these patients [27]. In our study, similar situations were observed. Furthermore, we observed 4 patients with plateaued or increased hsCRP levels after ATT; however, they all showed a more-or-less recovery pattern in colonoscopy. Therefore, hsCRP may not always be reliable. Colonoscopy is by far the most objective and reliable method, despite its limitations including the incapability of evaluation on most small intestine, high costs, potential complications, and restricted examination scope in case of bowel stenosis in some occasions. Therefore, more complete, comfortable, cost-effective methods to assess ATT responses are of utmost importance.

Cross-sectional imaging modalities, such as CTE, GIUS and magnetic resonance enterography (MRE), have been successfully used for assessing bowel lesions. For example, CTE is widely used for diagnosis, activity assessment and follow-up in Crohn’s disease (CD), a chronic inflammatory bowel disease that is highly analogous to ITB [28]. The intake of large volumes of neutral contrast agent, and the 3D reconstruction of the intestine shows a direct view of the overall bowel walls and extra-enteric lesions, such as abdominal lymph nodes, mesentery, and bowel supplying vessels [14]. GIUS is a non-radiated, non-invasive and cost-effective method with high sensitivity in detecting bowel lesions by evaluating thickened bowel walls, ulcers, intramural, and extramural abscesses, which are common manifestations in ITB [13]. Follow-up by imaging procedures may reduce the risks and high costs of colonoscopy, while giving a more thorough assessment of the small intestine. Disadvantages may include differences between different radiologists (also sonographers) to perform and read bowel images, which may largely diversify the quality of reports.

Reports on the utility of cross-sectional imaging techniques in diagnosis, assessment of activity, and follow-up monitoring in ITB are limited. The imaging features of ITB have been summarized in several studies [6, 14, 15, 21]. In several reports and a meta-analysis, the CT features for the differential diagnosis between CD and ITB is discussed [15, 25, 26, 29–33]. Generally, the CTE findings of ITB include: mural thickening, necrotic lymph nodes, ileocecal area involvement, fistulas and strictures. GIUS-related studies on ITB are rare [14, 34]. Common GIUS manifestations include: bowel wall thickening, bowel ulcers, hypoechoic bowel wall and abundant vascularity. Unfortunately, these imaging signs are generally non-specific. In our study, similar imaging signs as previously reported were observed, and they were carefully evaluated as our main criteria for ATT responses.

In our study, 3 patients received different evaluations from imaging and clinical evaluations. These discrepancies may be caused by the different viewing angles that CTE, GIUS and colonoscopy provide. CTE usually provides a panorama view, including lesions range, bowel wall, mural enhancement, and abdominal lymph nodes. GIUS is more focused, and provides more detailed information on the bowel wall structure, vascularity, and ileocecal ulcers. As ulcers can be clearly detected by colonoscopy from the mucosal surface, GIUS can assess the depth of the ulcer and surrounded complications, such as fistulas and intramural/extramural abscesses [14]. Colonoscopy can directly detect lesions from the inner mucosal surface of the intestine, and has the advantage that pathological specimens can be obtained. However, manifestations outside the intestinal lumen, including bowel wall and extra-intestinal features, are undetectable when using colonoscopy. As shown in Figs. 1 and 2, different information can be interpreted from the CTE/GIUS and colonoscopy regarding the same specific area. ITB is pathologically classified into 3 types: ulcerative, hypertrophic, and ulcerohypertrophic. Therefore, the pathophysiological disparities between these different types of ITB may result in unsynchronized healing within the bowel wall, thereby causing disagreement between different measurements. For example, one enrolled patient who was evaluated as having “partial response” by clinical evaluation and having “good response” by CTE after 3 months of ATT, was followed up for another 3 months. Data indicated that this patient showed significant progress in symptoms, hsCRP and colonoscopy. The re-evaluation as per clinical criteria should be “good response”. This suggested that cross-sectional imaging techniques can provide additional information or detect earlier signs when compared to clinical evaluation. Therefore, in our study, we proved the value of cross-sectional imaging techniques as an alternative or complementary approach to colonoscopy in follow-up assessment.

Our study had several limitations. First, the sample size of our study was small. This was limited by the lack of imaging follow-up data due to the paucity of literature and experience. Moreover, clinicians tend to use endoscopy and serological markers for follow-up. Not all patients received CTE and GIUS simultaneously because of economic reasons, available time, and CT radiation dose. We did not discuss the value of specific imaging signs in the evaluation of ATT responses, since there were not enough cases for each of the signs to make a conclusive statement. Therefore, future research is required to further validate the results that were obtained in our study.

## Conclusions

Reliable, non-invasive and repeatable evaluation of ATT responses in ITB is needed both for diagnostic and therapeutic purposes. Cross-sectional imaging techniques, including CTE and GIUS showed an outstanding accuracy when compared to the traditional colonoscopy-based clinical method. Large-scale studies are necessary to further validate the role of CTE and GIUS for ITB follow-up.

## Additional file

Additional file 1: The Additional file 1 includes the Supplementary Table 1–2. (DOCX 65 kb)

## Data Availability

The datasets used and/or analysed during the current study are available from the corresponding author on reasonable request.

## Abbreviations

AFB: Acid-fast bacilli
ATT: Anti-tubercular therapy
CD: Crohn’s disease
CTE: CT enterography
ESR: Erythrocyte sedimentation rate
GIUS: Gastrointestinal ultrasound
hsCRP: hypersensitive C-reaction protein
ITB: Intestinal tuberculosis
MRE: Magnetic resonance enterography
TB: Tuberculosis
