# Supplementary material for: The potential role of CT enterography and gastrointestinal ultrasound in the evaluation of anti-tubercular therapy response of intestinal tuberculosis: a retrospective study
Source: BMC Gastroenterol. 2019 Jun 26;19:106. doi: 10.1186/s12876-019-1030-0 (PMC6595613; doi:10.1186/s12876-019-1030-0)
Supplement: Supplementary file 1 — The Additional file 1 includes the Supplementary Table 1–2. (DOCX 65 kb) [file 12876_2019_1030_MOESM1_ESM.docx]

**Additional file 1: Table S1. The follow-up time and ATT course of recruited patients**

| **Patient No.** | **First follow-up time(month)** | **Total number of follow-up times** | **ATT duration**  **(month)** |
| --- | --- | --- | --- |
| **1** | 4 | 2 | 12 |
| **2** | 4 | 2 | 6 |
| **3** | 4 | 2 | 16 |
| **4** | 12 | 1 | 12 |
| **5** | 9 | 1 | 9 |
| **6** | 6 | 2 | 12 |
| **7** | 3 | 2 | 6 |
| **8** | 6 | 2 | 12 |
| **9** | 12 | 1 | 12 |
| **10** | 4 | 2 | 12 |
| **11** | 6 | 2 | 12 |
| **12** | 6 | 2 | 12 |
| **13** | 9 | 1 | 9 |
| **14** | 6 | 1 | 6 |
| **15** | 12 | 1 | 12 |
| **16** | 3 | 2 | 9 |
| **17** | 4 | 2 | 9 |
| **18** | 2 | 2 | 6 |
| **19** | 4 | 2 | 12 |
| **20** | 6 | 2 | 12 |
| **Average** | 6.1 |  |  |

**Additional file 1: Table S2. The comparison between CTE/GIUS and colonoscopy**

|  |  | Colonoscopy | |  | Kappa value* |
| --- | --- | --- | --- | --- | --- |
|  |  | Good response | Partial response | Total |  |
| CTE | Good response | 8 | 1 | 9 | 0.643  (P<0.01) |
|  | Partial response | 2 | 6 | 8 |  |
|  | Total | 10 | 7 | 17 |  |
| GIUS | Good response | 6 | 0 | 6 | 1  (P<0.01) |
|  | Partial response | 0 | 1 | 1 |  |
|  | Total | 6 | 1 | 7 |  |

* Kappa value: ≥0.75: excellent coincidence; 0.4-0.74: fair to good coindicence; ＜0.4: poor coincidenc
